# A Correlation Analysis between Undergraduate Students’ Safety Behaviors in the Laboratory and Their Learning Efficiencies

**DOI:** 10.3390/bs13020127

**Published:** 2023-02-02

**Authors:** Deng-Guang Yu, Yutong Du, Jiahua Chen, Wenliang Song, Tao Zhou

**Affiliations:** 1 School of Materials & Chemistry, University of Shanghai for Science & Technology, Shanghai 200093, China; 2 Shanghai Engineering Technology Research Center for High-Performance Medical Device Materials, Shanghai 200093, China

**Keywords:** course design, electrospinning, laboratory, learning efficiency, professional knowledge, quantitative evaluation, responsibility, safety behavior

## Abstract

Students’ behaviors have a close relationship with their learning efficiencies, particularly about professional knowledge. Different types of behaviors should have different influences. Disclosing the special relationship between undergraduate students’ conscious safety behaviors in their laboratory experiments with their learning efficiencies is important for fostering them into professional talents. In this study, a course entitled “Advanced Methods of Materials Characterization” was arranged to contain three sections: theoretical learning in the classroom, eight characterization experiments in the laboratory in sequence, and self-training to apply the knowledge. In the final examination, eighteen percent was allocated to the examination questions about safety issues. The students’ scores for this section were associated with their total roll scores. Two quantitative relationships are disclosed. One is between the students’ final examination score (*y*) and their subjective consciousness of safety behaviors (*x*) in their laboratory experiments, as *y* = 5.56 + 4.83 *x* (R = 0.9192). The other is between their grade point average (*y*) and safety behavior evaluation (*x*) as *y* = 0.51 + 0.15 *x* (R = 0.7296). Undergraduate students’ behaviors in scientific laboratories need to be verified to have a close and positive relationship with their professional knowledge learning efficiencies. This offers a hint that improving students’ safety behaviors and enhancing their subjective safety awareness are conducive to improving their learning efficiency for professional knowledge.

## 1. Introduction

During the growth of a person, suitable learning formats and their related learning efficiency are constantly changing. For the core fundamental knowledge that a student should grasp in primary and middle school, traditional classroom lectures are a traditional and effective manner for imparting knowledge. However, when the student is walking into university and beginning their professional knowledge study, learning formats should be gradually turned to practice education for becoming a talent in their future career [1,2]. 

For undergraduate students in university, particularly majoring in natural sciences and technologies, practice experiences are becoming more and more important, in addition to the theoretical professional knowledge learned in the classroom. Thus, for training the students’ practice capabilities, a series of practice courses is often arranged during their 2nd to 4th year. Among those practice courses, one useful type is the combination of theoretical knowledge in the classroom and practice training in a laboratory for a certain lecture. This has been demonstrated to be a highly useful method in many professional basic courses, such as “Materials Science and Engineering” and “Advanced Methods for Materials Characterizations”. 

The practice of laboratory experiments is a useful way to review and promote the subjective learning of professional knowledge in the classroom. How to carry out the practices is totally different in different disciplines. However, there is a common issue, i.e., the safety issue, which is particularly important for students majoring in natural sciences, particularly chemistry, biology, materials science, and engineering. It is well known that professional experiments in a scientific laboratory help to deepen students’ professional cognition [3,4]. However, few investigations can be found about the relationship between students’ behaviors (particularly those associated with their safety) and learning efficiencies. 

## 2. Literature Review

Some reports can be found about the relationships between primary school students’ behaviors and their special abilities, such as responsibility [5]. Further, some reports have correlated the relationship between students’ learning efficiencies and their behaviors in a special aspect, such as question-answering behavior [6]. What is more, even suitable humor is demonstrated to be useful for a positive educational purpose to impart knowledge to students [7]. Thus, it can be hypothesized that undergraduate students’ learning efficiencies about their professional knowledge may have a close relationship with their safety behaviors in laboratory experiments. 

During student growth, responsibility is always associated with their learning consciousness, and abundant reports have discussed that the enhancement of responsibility in many activities can be conducive to students’ learning results in the literature [8,9,10]. Particularly, some clear and definite tasks are very useful and often occur during the students’ school lives for strengthening their purpose of learning in addition to their basic ideals and beliefs [11,12,13]. Meanwhile, many new learning manners and phenomena have been disclosed in the positive relationship between responsibility and learning effect [14,15,16].

Although studies about the importance of safety experiments and how to ensure a safe environment for fostering student responsibilities and promoting their study interests are ample [17,18,19,20,21,22,23], investigations about the person safety issues (which include at least safety consciousness and safety behaviors) and students’ learning efficiencies cannot be found. Own safety is one of the most basic instincts of all people. It is obvious that own safety can be explored to effectively enhance students’ responsibilities [24,25,26,27], and, in turn, their learning effects from knowledge. This situation is even projected when students move to the professional knowledge learning stage, where some hazardous materials and experiments often appear in their learning and practices [28]. For example, Yu et al. recently analyzed a series of potential safety hazards that may occur in an electrospinning experiment in a scientific laboratory [29]. It is a pity that further applications of these hazards on promoting students’ safety responsibilities and the related learning efficiencies are seldom mentioned.

Based on the above-mentioned background, the present article investigated students’ safety behaviors and their learning efficiencies about a professional course, i.e., *Advanced Materials Characterization Methods*. Based on our knowledge, this is the first time this topic is put forward and is intentionally investigated. The course “*Advanced Methods of Materials Characterization*” is given in many disciplines in numerous universities and, thus, was selected as the targeted course. A reasonable arrangement of the lectures was designed for obtaining first-hand data to disclose the behavior–learning–efficiency relationships, which, in turn, give some hints on how to foster students into talented engineers.

## 3. Research Methods

### 3.1. Arrangement of the Course

**Figure 1:**
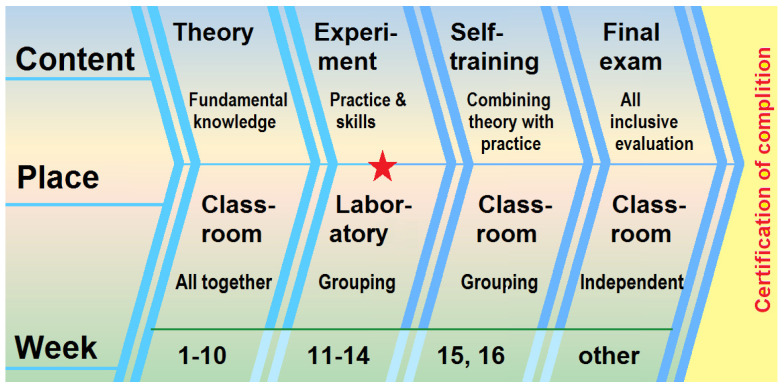
The content, place and time arrangements of the course “*Advanced Methods of Materials Characterization*” for Junior students.

The course of “*Advanced Methods of Materials Characterization*” is composed of three associated sections (Figure 1): theoretical learning in the classroom from the first week to the tenth week; eight characterization experiments in the laboratory for practices between the 11th and 14th weeks; and self-training for applying knowledge and training the capability of combining theory with practice. In the final examination for the certification of completion, 18 of the 100 points in the test are allocated to examination questions about safety issues. The laboratory behaviors of all the students can be reflected from the answers quantitatively. 

### 3.2. The Arrangement of the Laboratory Experiments

**Figure 2:**
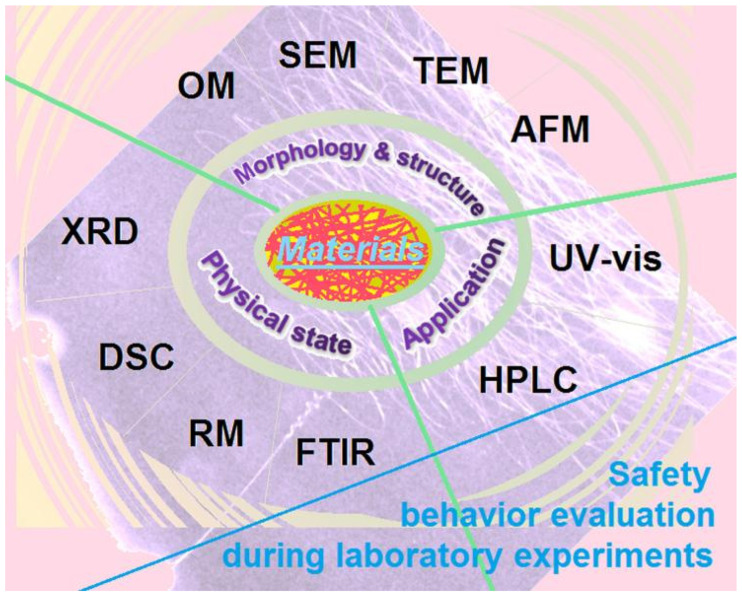
Eight experiments from the 10 theoretical lessons are arranged for the students to take part in the practice training.

The eight characterization experiments in the laboratory for practices between the 11th and 14th weeks are included in Figure 2. Two experiments, i.e., high-performance liquid chromatography (HPLC) and atomic force microscopy (AFM), were not arranged in the experiments although their theories had been given to the students in the classroom. The reason is that they cost too much time to finish the whole process. The other eight experiments were arranged in four weeks, two experiments for each week. The students are requested to conduct the experiments in groups.

These characterization methods can be divided into three groups. One group is to “see” things at the micro level, mainly morphology, shapes, and inner structures. These methods include optical microscopy (OM) [30,31,32], scanning electron microscopy (SEM) [33,34,35,36,37], and transmission electron microscopy (TEM) [38,39,40,41,42]. The second group is to evaluate the physical state of the components and their compatibility, through experimental methods, including roman spectroscopy (RM) [43], Fourier-transform infrared (FTIR) [44,45,46,47,48], differential scanning calorimetry (DSC) [49,50,51], and X-ray diffraction (XRD) [52,53,54]. The third group is an experiment about quantitative measurement, i.e., ultraviolet-visible spectroscopy (UV-vis) [55,56]. As shown in the references [30,31,32,33,34,35,36,37,38,39,40,41,42,43,44,45,46,47,48,49,50,51,52,53,54,55,56], these experimental methods are frequently and intensively exploited in scientific studies to evaluate composites and nanomaterials [57,58]. They are very useful for undergraduate students in terms of grasping fundamental knowledge and professional skills, and fostering the ability of working and innovation in their coming careers. 

In all the above-mentioned methods, the targeted materials were medicated fibrous membranes. These functional films were prepared using a top-down technique, i.e., electrospinning [59,60,61,62]. Electrospinning is a popular method for creating nanofibers [63,64,65,66]. It is a simple single-step process for solidifying polymeric solutions [67,68,69]. The drug can be facilely encapsulated into polymeric nanofibers through a blending solution of the guest drug and the host polymer [70,71,72,73]. Nonetheless, the mechanism of fibrous formation is extremely complicated [74,75,76,77,78,79]. Meanwhile, a series of safety issues is associated with the implementations of this method, such as electric shock and organic solvent [29]. Thus, great attention should be paid during laboratory preparation. In this study, both the electrospinning preparation and the characterizations of electrospun nanofibers are conducted in laboratories. The students are reminded to take care of their behaviors during the safety education in the classroom.

### 3.3. The Evaluation in the Final Examination

The safety issue questions have three sub-problems, with a total 18 points as follows.

**Figure 3:**
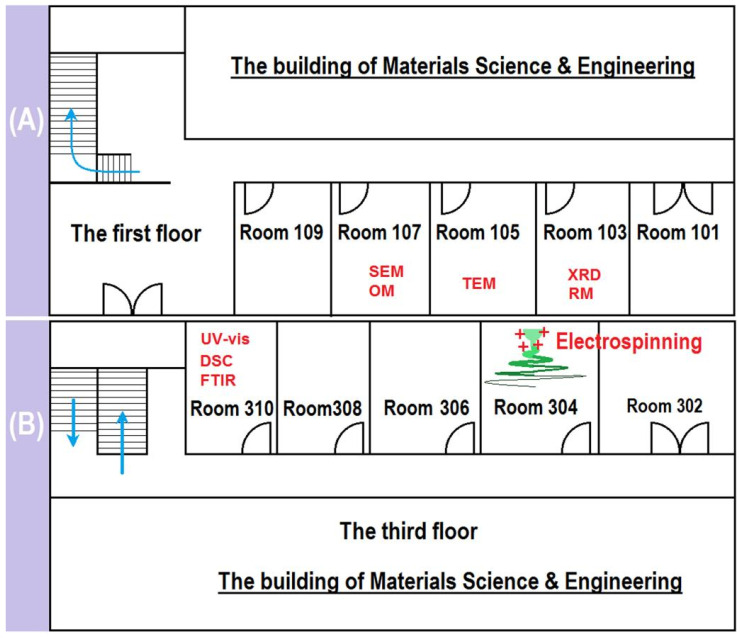
The places for carrying out the 8 characterization experiments and the preparation of drug-loaded nanofibers: (**A**) the first floor of the Materials Building in University of Shanghai for Science and Technology; (**B**) the third floor of the Materials Building (the answers are written in the blank).

1. In the experimental lessons, we conducted eight experiments in different rooms. These experiments include: OM, SEM, TEM, RM, and XRD (Figure 3A); FTIR, DSC, UV-vis, and electrospinning preparation (FTIR was directly given in the blank) (Figure 3B). Fill the experiments in their located rooms, where they were carried out (7 points).
2. Give the full names of OM, SEM, TEM, RM, FTIR, DSC, XRD, and UV-vis. (4 points). The standard answers are: OM—Optical microscopy (or microscope); SEM—Scanning electron microscopy (or microscope); TEM—Transmission electron microscopy (or microscope); RM—Raman spectroscopy; FTIR—Fourier-transform infrared; DSC—Differential scanning calorimetry; XRD—X-ray diffraction; and UV-vis—Ultraviolet-visible spectroscopy.
3. Which one of those rooms is the most dangerous place for you? Describe your behaviors during the experiments and point out the possible unsafe factors (7 points, no standard answer).

### 3.4. The Data-Treating Methods

The students’ scores of the safety section are analyzed through association with their total roll scores, and also their grade point average (GPA) values during their past two and a half years. A linear equation is exploited to treat the data using Origin 8.5. The samples are thirty students in a natural class from two “Material Science and Engineering” and “Material Forming and Control Engineering” majors at the School of Materials and Chemistry in the University of Shanghai for Science and Technology. 

## 4. Results 

The Scores of the Safety Question and the Final Examination

**Figure 4:**
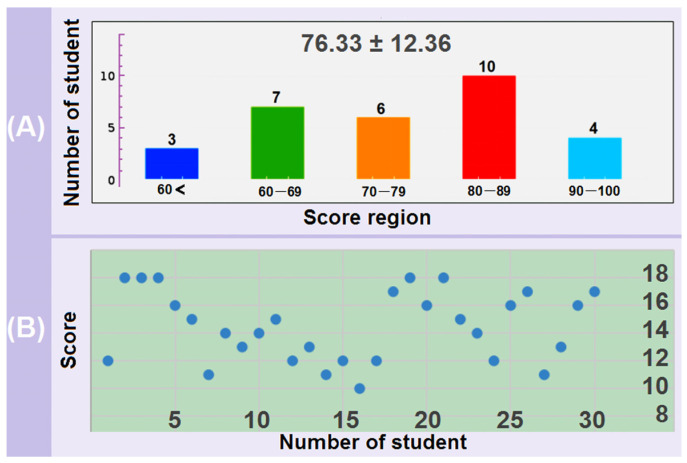
The distribution of scores of the whole final examination (**A**) and the safety issue questions (**B**) from the 30 graduate students in the class.

The scores of the whole examination, the safety issue scores, and the GPA values of the thirty Junior undergraduate students are included in Table 1. The pass rate is 90%, with the lowest score being 50 and the highest score 95. The average grade of the whole class is 76.33 ± 12.36, as indicated in Figure 4A.

As for the safety issue questions, the scores are between 10 and 18 points, with five students obtaining the full 18 points. The scattering plot of these scores is drawn in Figure 4B. Several students lost their scores in the second sub-question due to grammar mistakes for professional words. The main differences are from the students’ behaviors about the first and third sub-questions. 

## 5. Discussion

During the learning process, the students’ active participation can effectively improve their learning efficiency. The active participation can be reflected through certain behaviors. In the learning of professional knowledge, both classroom participation and laboratory participation are closely related to students’ subjective learning. Safety behavior and related safety awareness are some of the most basic and important parts of practical behaviors in the laboratory. Therefore, theoretically, there is a definite relationship between safety behavior and the professional knowledge learning effect. This study is intentionally carried out to disclose this internal relationship.

### 5.1. The Validation of the Arrangement of the Course

No matter how to reform the class for effective teaching, the rules of the Academic Affairs Office of the University of Shanghai for Science and Technology must be obeyed. According to the regulations, the pass rate of all the students and the average scores of final examinations should be over 80% and 70, respectively. Provided these standards cannot be met, the course is too difficult for the students. Then, the difficulty of professional learning should be reduced. Here, a pass rate of 90% and an average grade of 76.33 ± 12.36 (Table 1 and Figure 4A) suggested that the teaching reformation of three sections was valid. Meanwhile, the scores of the final examination and also the safety issue are typical Normal Gaussian distributions, giving a hint that the evaluation results of the safety behaviors and learning efficiencies are consistent with the conventional expectations. 

### 5.2. The Safety Questions Reasonably Reflect the Students’ Behaviors

The first sub-question about which room is the experimental place seems very easy, but it can exactly reflect the consciousness of the students’ own behaviors. Some of the students went into the laboratory following their classmates, without a sober consciousness of the room number. The third sub-question goes deeper, from personal safety awareness to personal safety behaviors. From the column of “Scoring rate of the 18 points” in Table 1, which is the percentage of the students’ scores about the 18-points safety questions, the values are always larger than the interval average values in the third column in Table 1, i.e., the “Mean” of final examination. This situation suggests that the students achieved a relatively higher score here than the theoretical questions. In the column of “Proportion to total score” (Table 1), with the ratio of absolute safety question score to the absolute total final examination score, the values are always slightly larger than the value of 18. This phenomenon similarly reflects that the safety issue question contributed roles in keeping a final valid and reasonable final examination.

### 5.3. The Relationship Analyses 

**Figure 5:**
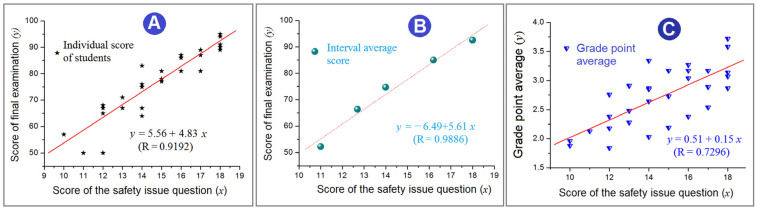
Analyses of the several relationships with score of the safety issue question: (**A**) the scores of whole final examination (in individual manner); (**B**) the scores of whole final examination (in interval average manner); (**C**) the grade point average of all the class students.

To analyze the relationship between the scores of safety questions and whole final examination, two methods are conducted. One is from all the individual students, and the other is from the interval average score. Their results are shown in Figure 5A,B, respectively. 

The regressed equation for the score of the whole final examination (*y*) to the score of the safety issue question (*x*) in an individual manner in Figure 5A is *y* = 5.56 + 4.83 *x*, which has a correlation coefficient R of 0.9192. This value suggests that students’ learning efficiencies have a close relationship with their safety behaviors in the laboratory. Furthermore, the regressed equation for the score of the whole final examination (*y*) to the score of the safety issue question (*x*) in an interval average manner in Figure 5B is *y* = −6.49 + 5.61 *x*, which has a correlation coefficient R of 0.9886. This value further indicates the positive relationship between students’ learning efficiencies with their safety behaviors in the laboratory.

The students’ GPA values (y) are further associated with their safety issue evaluation results (x). Despite a weaker correlation coefficient R of 0.7296, there is still a positive relationship between them, i.e., *y* = 0.51 +0.15 *x* (Figure 5C). This smaller R value can be explained in that the GPA values show mainly the learning effect of basic courses during their freshman and sophomore years, such as advanced mathematics, college physics, college chemistry, and English. This concern also shows several limitations of the present investigations, including the small size of the sample (only 30 students participated in the study) due to the limitation of teaching resources, not having a control group with only theoretical lessons in the classroom, and the performances of these students in future professional courses. Further studies will be carried out in the next year and the year after. 

## 6. Conclusions

To disclose the relationship between the students’ safety behaviors in the laboratory and their learning efficiencies for professional knowledge, traditional classroom teaching was reformed based on the course of “*Advanced Methods of Materials Characterization*” for Junior students. Three sections, consisting of classroom theoretical learning, experiments in laboratories, and self-training, were set up for obtaining data and also for training the students’ capabilities of practices and the combination of theoretical knowledge with practice. Meanwhile, the final examination was revised to contain safety issue questions, which reflected their behaviors in the laboratories. The students’ scores of the safety section are analyzed through association with their total roll scores using a linear regressed equation; a positive relationship between the safety behaviors (*x*) and whole lesson learning efficiencies (*y*) is disclosed as *y* = −6.49 + 5.61 *x* with a high correlation coefficient of 0.9886. The disclosed positive relationship is useful for improving teaching methods, enhancing the students’ learning consciousness, and effectively implementing the “three-whole education” in higher education institutes advocated by the Ministry of Education of China. 

## 7. Suggestions for Practical Use

From the above-mentioned results and discussion, the following useful hints can be considered for improving students’ learning efficiencies about professional knowledge.

1. For the lessons of basic professional courses, a combined curriculum can be arranged for undergraduate students, which can contain theoretical lessons in the classroom, practice training in the laboratory, and also self-training lessons.
2. Students often prefer information related directly to the target experiments [80]. When they personally experience safety issues in the laboratory, their learning effects about professional knowledge will be augmented. Reasonable selections of the experiments are important.
3. The students’ whole final examination scores have a linear relationship with their safety question scores, both in an individual manner and in an interval average manner. Safety education and evaluation can be useful tools for promoting the teaching of responsibility and also elevating learning effects.
4. Improving students’ safety behaviors and enhancing their subjective safety awareness are conducive to improving learning efficiency for professional knowledge.
5. Certainly, for effectively implementing safety education about professional practices, it is important that teachers should try their best to follow the most recent developments in their scientific fields [9]. Only based on this point, students can be imparted with cutting-edge techniques and professional knowledge. Meanwhile, online learning platforms have become a universal medium for knowledge acquisition and online teaching because of the influence of the COVID-19 pandemic in the last three years [81]. This situation would make professional practices, safety education, and related behaviors even more important for students to become a talent in their future work.

## Figures and Tables

**Table 1:** All the students’ scores of the whole examination (from highest to lowest scores) and safety issue, as well as their GPA values.

Student No.	Final Examination		Safety Issue Question		Ratio (%)		GPA ^a^
	Individual	Mean	Individual	Mean	Scoring Rate of the 18 Points	Proportion to Total Score	
1	95	92.5	18	18.0	(18/18) × 100% = 100	(18/92.5) × 100% = 19.5	3.14
2	94		18				3.58
3	91		18				2.87
4	90		18				3.72
5	89	85.0	18	16.2	(16.2/18) × 100% = 90.0	(16.2/85) × 100% = 19.1	3.07
6	89		17				3.17
7	87		17				2.54
8	87		16				2.38
9	86		16				3.27
10	86		16				3.17
11	83		14				3.34
12	81		17				2.89
13	81		16				3.04
14	81		15				2.73
15	78	74.7	15	14.0	(14/18) × 100% = 77.8	(14/74.7) × 100% = 18.7	2.19
16	77		15				3.17
17	76		14				2.87
18	75		14				2.64
19	71		13				2.28
20	71		13				2.48
21	68	66.4	12	12.7	(12.7/18) × 100% = 70.6	(12.7/66.4) × 100% = 19.2	2.18
22	67		14				2.85
23	67		13				2.91
24	67		12				2.76
25	67		12				2.38
26	65		12				1.84
27	64		14				2.03
28	57	52.3	10	10.3	(10.3/18) × 100% = 57.2	(10.3/52.3) × 100% = 19.7	1.88
29	50		10				1.96
30	50		11				2.13

^a^ GPA is an abbreviation of grade point average, referring to students’ learning efficiencies during the past 5 semesters in their college life with a full value of 5.

## Data Availability

The data supporting the findings of this manuscript are available from the corresponding authors upon reasonable request.
